# Application of imaging and spectroscopy techniques for grading of bovine embryos - a review

**DOI:** 10.3389/fvets.2024.1364570

**Published:** 2024-05-07

**Authors:** Manickavasagan Shivaani, Pavneesh Madan

**Affiliations:** Department of Biomedical Sciences, Ontario Veterinary College, University of Guelph, Guelph, ON, Canada

**Keywords:** embryo, morphokinetics, machine learning, morphological features, FTIR, Raman spectroscopy

## Abstract

Although embryo transfers have grown considerably in the cattle industry, the selection of embryos required for successful pregnancies remains a challenging task. Visual inspection of 7th-day embryos using a stereomicroscope, followed by classification based on morphological features is the most commonly practiced procedure. However, there are inaccuracies and inconsistencies in the manual grading of bovine embryos. The objective of this review was to evaluate the potential of imaging and spectroscopic techniques in the selection of bovine embryos. Digital analysis of microscopic images through extracting visual features in the embryo region, and classification using machine learning methods have yielded about 88–96% success in pregnancies. The Raman spectral pattern provides valuable information regarding developmental stages and quality of the embryo. The Raman spectroscopy approach has also been successfully used to determine various parameters of bovine oocytes. Besides, Fourier Transform Infrared (FTIR) spectroscopy has the ability to assess embryo quality through analyzing embryo composition, including nucleic acid and amides present. Hyperspectral Imaging has also been used to characterize metabolite production during embryo growth. Although the time-lapse imaging approach is beneficial for morphokinetics evaluation of embryo development, optimized protocols are required for successful implementation in bovine embryo transfers. Most imaging and spectroscopic findings are still only at an experimental stage. Further research is warranted to improve the repeatability and practicality to implement in commercial facilities.

## 1 Introduction

The embryo transfer technology in bovine was introduced commercially in 1970’s. Currently, the selection of embryos to be used for *in-vivo* produced multiple ovulation and embryo transfer (MOET) or *in vitro* produced (IVP) is carried out by specialized embryologists. In many places, there is no specified embryologist available always for evaluation and grading of embryos. The selection of embryos for transfer is a critical step in the successful breeding of cattle. The morphological features that are most commonly followed to grade embryos are explained by the International Embryo Technology Society (IETS). IETS defines the criteria for the quality of embryos and developmental stages through a numerical grading system: Category I- Stage of development: 1-unfertilized oocyte or a 1-cell embryo; 2–4 cell embryo; 3- morula (mass of at least 16 cells); 4-compact morula; 5-early blastocyst; 6-blastocyst; 7-expanded blastocyst; 8-hatched blastocyst; 9-expanding hatched blastocyst Category II- Quality of embryo based on morphological features: 1-Excellent or good; 2-Fair; 3-poor; 4-dead or degenerating.

### 1.1 Grading methodology

Bó and Mapletoft (1) explained the process to assess bovine embryos using IETS protocols. The assessment is generally carried out using a stereomicroscope (50-100X) while the embryo is on a dish. It is recommended to roll the embryo in order to view the zona pellucida at different angles. The mean diameter of the bovine embryo ranges from 150 to 190 μm, and thickness of zona pellucida ranges from 12 to 15 μm. The diameter of the embryo is consistent from single cell to blastocyst stages. The desired morphological features for an ideal embryo are: compact and spherical; blastomeres - similar size, even color and texture; cytoplasm - should not be granular or vesiculated; perivitelline space - clear and no cellular debris; zona pellucida - uniform, neither cracked nor collapsed, no debris on its surface.

### 1.2 Current success rate with bovine embryo transfers

There is a direct relationship between embryo quality and post-embryo transfer gestational success (2, 3). In other words, embryos that are morphologically distinguished to be a higher rank (grade 1) have greater success rates during gestation. The selection of an ideal embryo for transfer, and its consequent gestational success has been an ongoing challenge. The current selection process through manual methods have yielded only a 30–50% success rate in pregnancy even with good to excellent grade embryos (4, 5). Further, most of the time it is difficult to have data with transfer of all quality embryos, and mostly it is limited to excellent to good grade.

### 1.3 Challenges in manual selection of bovine embryos

Although there are several guidelines available for embryo classification, great subjectivity exists in the real-time decision-making processes which leads to grading variations. The manual method is subjective and biased based on the experience of the evaluator and other environmental factors. Farin et al. (6) proved that a given embryo was assigned to different grades when analyzed by different examiners. Furthermore, it was also observed that consecutive evaluations by the same evaluator yielded different grades for the same embryo (no repeatability). While evaluating the stage of development and quality of 40 bovine embryos, Farin et al. (6) studied the agreement between six evaluators. Although 89% agreement was achieved on the stage of embryo development, only 69% agreement was obtained among the examiners regarding quality ranking. The variable success rate of transferred embryos in MOET and IVP is one of the limitations of bovine embryo technology, as it requires a surplus of recipient cows which increases the overall cost per calf and associated farm logistics. Thus, it is necessary to develop an objective, automated, accurate, and fast method to determine the viability of embryos to ensure success in their subsequent implantation and gestation.

The current developments in machine learning techniques provide opportunities to analyze images and videos to make objective decisions for embryo grading. These techniques utilize visual variables present in morphokinetic imaging, alongside biological features of a developing embryo to determine the success of an embryo to complete its development and gestation period. The objective of this review is to evaluate the status and challenges in the development of image or spectroscopy based tools to grade and assess bovine embryo quality using machine learning models for automatic prediction of embryo quality to improve the outcome of pregnancy success rate in embryo transfer technology program.

In the last decade, enormous amount of work has gone into morphokinetic research using time-lapse imaging technique in analyzing human embryos (7–9). However, this technology is still in infancy for bovine embryos. So far, optical microscopy, Raman spectroscopy, Fourier Transform Infrared (FTIR) spectroscopy and near infrared (NIR) hyperspectral imaging have been tested for their potential to assess bovine embryo quality.

## 2 Optical microscopic imaging

The optical microscope uses visible light region of the electromagnetic spectrum (transmission or reflection) and appropriate lenses to magnify the small objects. In general, these microscopes are less expensive and simple to operate when compared to other techniques. The microscopic images can be automatically analyzed to obtain useful information for decision making.

### 2.1 Image segmentation

Image segmentation is a process in which the region of interest (ROI) from any image is separated from the background to obtain useful information. The selection of segmentation methods and procedures must be accurate in order to avoid background noise or losing valuable information from the ROI. The selection of a suitable segmentation technique requires preliminary experimentation with target images (10). Melo et al. (11) developed algorithms for the automated segmentation of bovine embryos. After preprocessing, a thresholding technique was implemented based on statistical features. The images of 30 embryos (early cleavage, morula, and blastocyst stages) were segmented and accuracy was then compared with gold-standard reference images. They achieved 91–93%, 93–96 and 92% segmentation accuracy for early cleavage, morula, and blastocyst stages of embryos, respectively.

### 2.2 Machine learning techniques for classification of embryos

The machine learning (ML) techniques develop algorithms to learn from dataset and used for further classification or prediction of unknown data without additional programming (12). The learning or training process in ML techniques may be broadly classified into supervised, unsupervised and reinforcement. In supervised learning, the learning for a model is from “labeled training data” that helps in making classification or prediction about the future data. In this learning process, the labeling of data is done to guide the machine to look for the pattern. The examples for the supervised learning tools are Artificial Neural Network (ANN), Decision Trees, Random Forest, Support Vector Machines k-Nearest Neighbor, Logistic Regression, Naïve Bayes, Linear Discriminant Analysis, Quadratic Discriminant Analysis and so on. Deep learning (DL) is a subset of ANN which involves a representation-learning through utilization of the deep ANN with multiple neuron layers. Convolutional neural network (CNN) is the commonly used DL for analyzing images. Unsupervised ML uses unlabeled data or unknown data structure for training. It analyzes the data structure to obtain meaningful information without the help of a known outcome variable. The examples for unsupervised ML tools are k-means Clustering, Independent Component Analysis (ICA), Principle Component Analysis (PCA) and so on. In reinforcement learning, the specific task-oriented algorithms are developed to learn and achieve a complex goal.

de Souza Ciniciato et al. (13) designed a smartphone-based user interface to classify bovine embryos in accordance with IETS grades. A smartphone was attached to the eyepiece lens of a stereomicroscope to perform the real-time evaluation. An ANN model with 482 bovine blastocyst images was developed by Rocha et al. (14) to use in the smartphone based classification of embryos. They also developed an online server^1^ that can be used through a phone or computer to perform embryo evaluation.

In another study, Matos et al. (15) developed digital image-based classification system for bovine blastocysts. Morphological features of the embryos were used to build ANN classifier. They tested 56 embryos (15% of the total images) using the developed model. The system took 2.5 s per image to complete the tasks (image uploading, preprocessing, feature extraction and classification), and provide a grade in accordance with the IETS classification. The accuracy of the ANN system was 84, 20 and 89% for grade 1, grade 2 and grade 3, respectively. They observed poor classification in grade 2 as they were misclassified as grade 1 or grade 3. They recommended using other imaging techniques or ML approaches to builda robust model and improve the classification efficiency.

Another automated embryo classification model was developed with 482 images of *in vitro* produced bovine blastocysts by Rocha et al. (16). An inverted microscope (32X magnification) was used to obtain images (one embryo per image). The image dataset had 113 grade − 1, 175 grade − 2 and 194 grade − 3 embryos as confirmed by three experienced embryologists as per the IETS standards. The developed ANN model classified the embryos with an accuracy of 76 to 88%.

Thompson (17) developed a prediction model for embryo quality to determine the success in the embryo transfer program. They used 476 inverted microscopic images of day 7 embryos (MOET pregnant −266; MOET non-pregnant-100; JIVET (Juvenile animals) pregnant-62; JIVET non-pregnant-63) from different Australian breeds - Angus, Poll Hereford, Wagyu and Composite cattle. A model was developed using the top 10 contributing features from the microscopic images. The pregnancy or non-pregnancy was confirmed on day 60. The model yielded up to 88% for MOET embryos and 96% for JIVET embryos.

The implementation of advanced image enhancement, image preprocessing, image analysis and ML-based classification models have the potential to build robust and objective and accurate grading systems with existing optical microscopes.

## 3 Time-lapse imaging

*In situ* monitoring of a developing embryo inside an incubator using video or time-lapse imaging is another approach for determining embryo quality. In this method, the embryo does not experience any culture-related stresses. Advancements in video analysis provide a plethora of opportunities to accurately evaluate embryo development using morphokinetic observations (18, 19).

The first time-lapse monitoring for bovine embryo was carried out by Grisart et al. (20) in 1994 using an inverted microscope (21). They monitored 130 embryos from one-cell stage to blastocyst stage for 8 days and observed timing of cleavage, duration of each cell cycle, time to blastocyst stage. It was reported that faster the embryos cleaved during the early stages, the higher its ability to develop to blastocyst stage.

Sugimura et al. (5) used time-lapse cinematography (TLC) to observe numerous prognostic factors including timing of the 1^st^ cleavage, number of blastomeres at the end of 1st cleavage, presence of multiple fragments at the end of 1st cleavage, number of blastomeres at the onset of the lag-phase, and oxygen consumption at the blastocyst stage using a real-time cultured cell monitoring system (CCM-MULTI, Astec, Japan). Around 673 images were taken during a 168 h culture period at 15 min intervals (4X magnification) and analyzed. This approach yielded 79% accuracy in pregnancy prediction.

**Figure 1:**
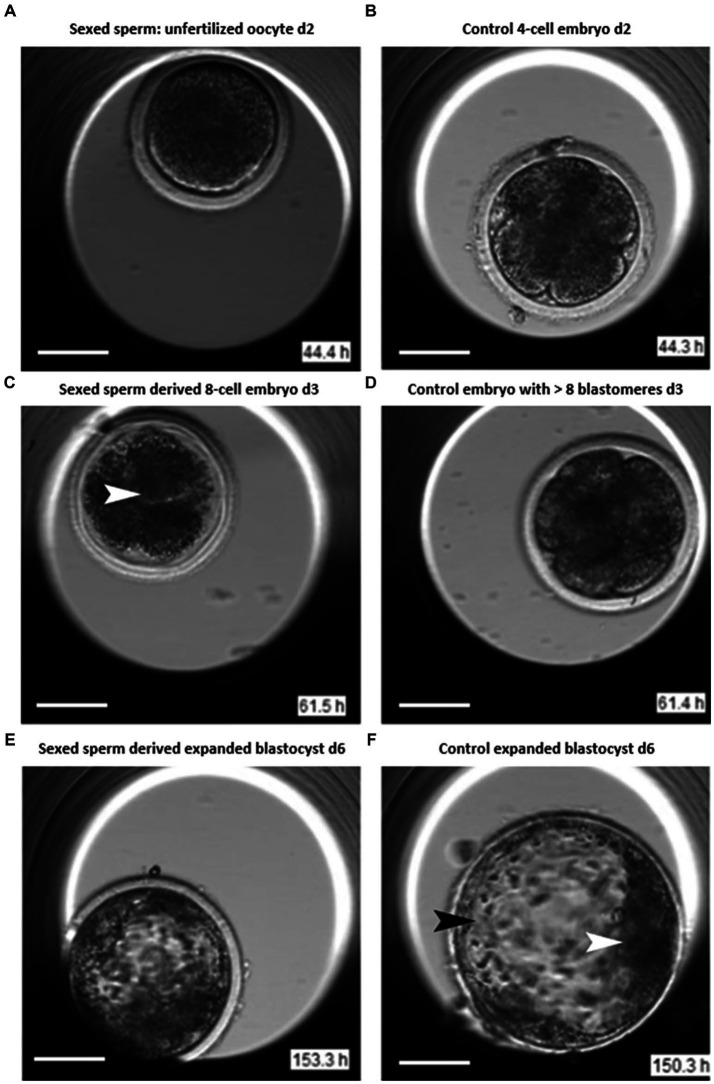
Images of sexed and conventional early embryos (22). Day 2: **(A)** Unfertilized oocyte displaying cleavage failure after insemination. **(B)** Control 4-cell embryo after insemination. Day 3: **(C)** Sexed sperm derived embryo displaying blastomere lysis (arrow). **(D)** Control embryo revealing >8 blastomeres. Day 6: **(E)** Sexed sperm derived expanding blastocyst. **(F)** Conventional sperm derived expanded blastocyst with trophectoderm cells (black arrowhead) and inner cell mass (ICM, white arrowhead).

The differences in the early stages of embryo development between sex-sorted and un-sorted (control) bovine sperm were investigated by Steele et al. (22) using morphokinetic investigation with the help of time-lapse imaging and video microscopy techniques. In this study, cryopreserved semen from Holstein Friesian bulls were used for *in vitro* fertilization. A time-lapse video-microscopy was used to record and analyze the development of 360 embryos. It was reported that the embryos derived from sexed-sperm showed increased incidences of arrest at the 4-cell stage. The embryos derived from sexed-sperm were at a higher risk for shrinkage/fusion of blastomeres with subsequent lysis resulting in reduced blastocyst rates (Figure 1). Further, embryos derived from sex-sorted sperm had a lower chance of cleavage and reduced survival times than that of conventional sperm. However, there was no difference in total time required for completion of development of embryos in both sex-sorted and conventional sperms. It was concluded that sexed-sperm kinetics were more discordant than conventional sperm kinetics, and time-lapse video-microscopy is a useful tool for consistently monitoring embryos.

Magata (21) mentioned that continuous observation of bovine embryo with the help of time-lapse monitoring techniques will yield accurate quantification of cellular dynamics and cell cycle length. This method has the capability to predict ploidy status of embryos because morphokinetics patterns are different for normal and aneuploid embryos due to aberrant chromosome complement.

Magata et al. (23) investigated the growth potential of bovine embryos showing abnormal cleavage during early developmental stages (reverse cleavage-RC; direct cleavage-DC), irregular and unsmooth ruffling of the oolema membrane (ruffling) through time lapse imaging. For each embryo, *in situ* images were taken at 20 min intervals for 240 h. They observed that about 36% of embryos that developed into a blastocyst showed abnormal cleavage. The morphokinetic investigation explained that RC, DC, and ruffling embryos showed slower development than that of normal cleaved embryos. The Embryos with RC and DC showed impaired hatchability with increased collapse of the blastocyst cavity until hatching. Also, it was reported that RC and DC embryos had increased chromosomal aneuploidy.

Since limited morphokinetic knowledge is available for the bovine embryo development, more research is required in this field to develop accurate techniques for commercial applications.

## 4 Raman spectroscopy

Alongside the use of morphological features for the assessment of developmental stages and bovine embryo quality using color imaging or optical microscopy, there are several other techniques being researched to support objective embryo grading. The principle, merits and demerits of each technique that are used for embryo quality assessment are given in Table 1. Cofactors other than morphological features such as timing of first embryonic cell divisions or metabolic profiles may be used to predict the ability of an embryo to establish pregnancy or as markers of embryonic viability (27). In general, the metabolomics profile of embryos is not yet established for bovine subjects.

**Table 1:** Comparison of imaging and spectroscopic techniques.

Technique	Principle	Merits	Demerits	Reference
Color/optical imaging	-Visible light (wavelength range: 400–700 nm) is transmitted through or reflected from the samples and captured by detectors to form an image -In microscopes, single or multiple lenses are used to obtain the magnified view of the sample	-Low to medium initial investment -Good tool to characterize surface or morphological features of sample - No damage to live cells	-May not be a good tool to characterize the internal information or chemical composition of samples	(24)
Raman spectroscopy / imaging	-The sample is exposed to laser energy at wavelengths in the near infrared (NIR), visible, or ultraviolet ranges -The molecules inside the object get vibrated and a vibrational spectrum is produced (based on scattered light of vibrating molecules) -Each molecule in the sample has a distinct set of nuclear vibrations, and hence the object as a whole has a distinct vibration-generating signature or molecular barcodes with high information content	- Provides qualitative and quantitative information of samples based on chemical composition - little or no sample preparation is required (low cost glass containers are ideal in most cases)	-Medium to High initial investment -Some region of the sample may be destroyed based on the intensity of the laser light -Complex analysis required	(25)
Fourie Transform Infrared (FTIR) spectroscopy	-The infrared light is split by the interferometer, and then directed to the sample -The sample absorbs some energy and transmits or reflects some energy based on chemical property -The detector receives the light from the sample and yields the spectrum	-High signal-to-noise ratio and scan speed	-Medium to High initial investment -Complex analysis required	(26)
Hyperspectral imaging	-The sample is imaged over a large number of spectral bands (wavelength range: 400–2,500 nm) and yields complete reflectance spectrum with spatial information (images) and spectral information	-Provides large data set with complete information about intrinsic properties and extrinsic characteristics of sample	-High initial investment -More time required to capture and analyze the images -Complex analysis required	(26)

**Figure 2:**
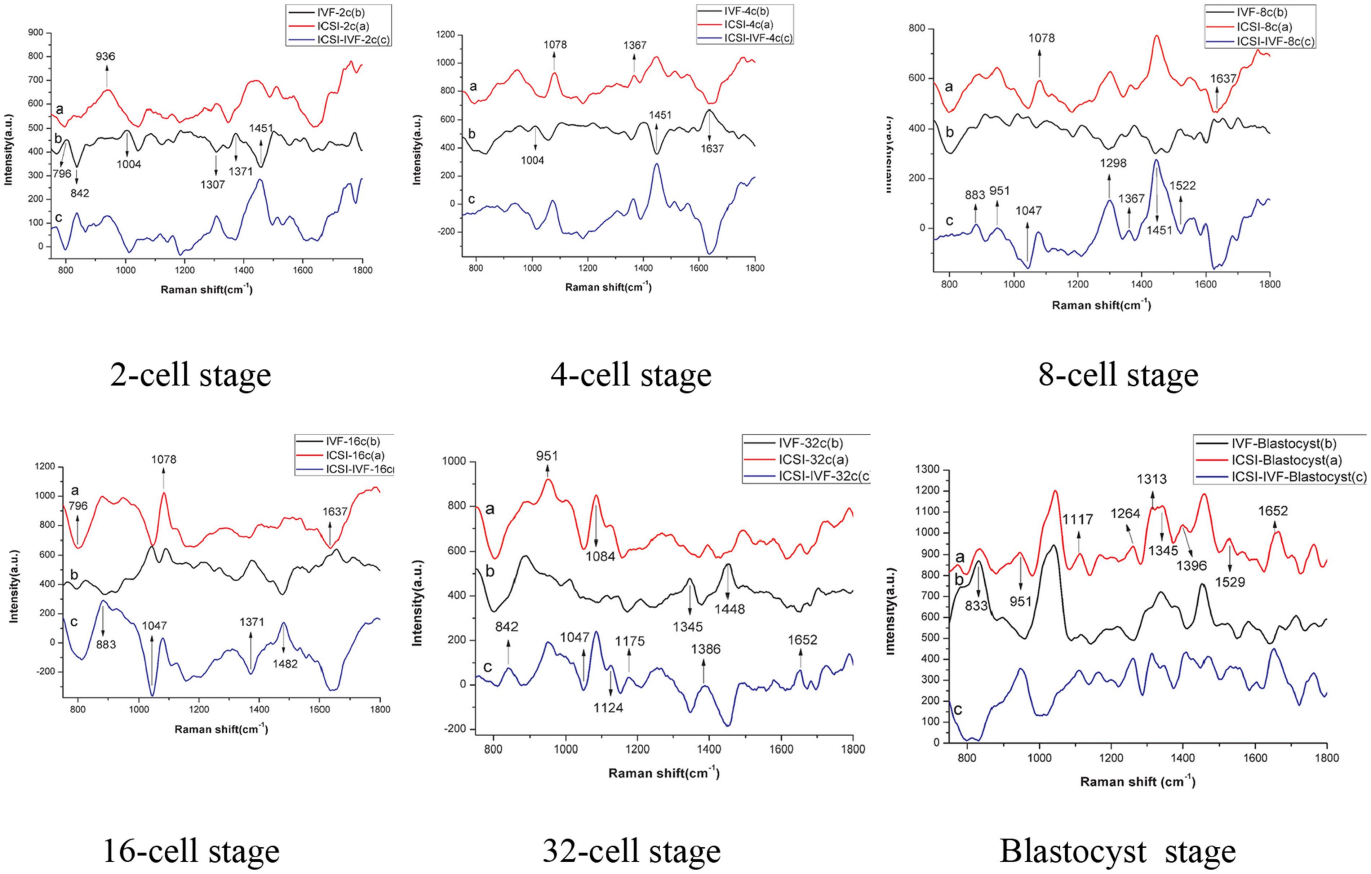
Raman spectra of culture medium: a - intracytoplasmic sperm injection (ICSI) b - *in vitro* fertilized (IVF) c - difference spectra between ICSI and IVF (28).

Laser tweezer Raman spectroscopy (LTRS) was used to evaluate the differences in composition between the embryo culture media of *in vitro* fertilized (IVF) and intracytoplasmic sperm injection (ICSI) methods (28). The bovine embryos were produced through ICSI and IVF methods to the 2-, 4-, 8-, 16-,32-cell, and blastocyst stages with individual *in vitro* culturing, and the culture media for embryos at different developmental stages were analyzed using LTRS. It was reported that the composition of culture media between IVF- and ICSI-derived embryos differed in carbohydrates, lipids, DNA, and proteins. The wave bands had specific patterns at 1004 cm^−^1 (phenylalanine) and 1,529 cm^−^1 (-C=C-carotenoid) which were associated with metabolic activity of embryos (Figure 2).

Raman microscopy has the potential in determining the quality of bovine oocytes. This technique may be used as a screening tool for selection of oocytes for fertilization. Jimenez et al. (29) measured the biochemical changes in the cytoplasm of bovine oocytes during the *in vitro* maturation process using Raman microscopy. The markers for proteins, lipids, and carbohydrates were used to investigate the ooplasm at four stages. It was reported that lipid accumulation was higher in the first 6 h of culture, carbohydrates were decreased during the developmental stage, and protein content reached average levels characteristic of mature oocytes within the last 4 h of the maturation process. In another study, Rusciano et al. (30) investigated the vitrification-induced structural damage in bovine oocytes. Raman microspectroscopy was used to quantify the biochemical variations in zona pellucida and cytoplasm of vitrified in cryogenically preserved vitro-matured bovine oocytes at different post-warming times. It was found that vitrification induced a transformation of the protein’s secondary structure from α-helices to *β*-sheet form. It was also observed that lipids tend to assume a more packed configuration in the zona pellucida. A decrease in lipid unsaturation was measured in vitrified oocytes (may be due to oxidative damage).

## 5 Fourier transform infrared (FTIR) spectroscopy

Wiechec et al. (31) used Fourier transform infrared (FTIR) spectroscopy and focal plane array (FPA) imaging to determine the quality of bovine embryos based on nucleic acids and amides. Three different blastocysts were used for this study: (1) Blastocyst 1 (BL1-HA) - fertilized oocyte cultured with low concentration of hialuronian (HA). (2) Blastocyst 2 (BL2-SOF) - oocytes cultured in standard conditions and cultured in SOF medium. (3) Blastocyst 3 ((BL3-VERO) - oocytes cultured in standard conditions and Cleavage stage blastocyst) cultured in co-culture with VERO cells. The FTIR spectrum of the inner cell mass of the single blastocysts was collected for this study. The differences in DNA were analyzed between wavenumbers 1,240 and 950 cm^−^1 and for protein amides was analyzed between wavenumbers 1800 and 1,400 cm^−^1 using principal component analysis (PCA) and Hierarchical Cluster Analysis (HCA). The multivariate statistical analysis (Hierarchical Cluster Analysis – HCA and Principal Component Analysis – PCA) of single cells spectra showed a high similarity of cells forming the inner cell mass within a single blastocyst. It was reported that the difference between the three types of blastocysts was significant in amide bands, and the quantitative and qualitative composition of the protein can be used to examine blastocysts. They concluded that FTIR spectroscopy has potential in reproductive biology for quality estimates of bovine embryos at various developmental stages, however further research is warranted to validate these approaches.

The investigation of zona pellucida protein during fertilization and pre-implantation might yield valuable information about the quality of oocytes or embryo. Nara et al. (32) used FTIR to investigate the secondary structure of zona during fertilization. It was found that attenuated reflection-FTIR spectrum of intact bovine zona pellucida was different between before fertilization and after fertilization (blastocyst stage). The ß-structure content was increased during fertilization. It was reported that changes in zona architecture during fertilization was due to changes in the secondary structure of the zona protein.

## 6 Hyperspectral and multispectral imaging

**Figure 3:**
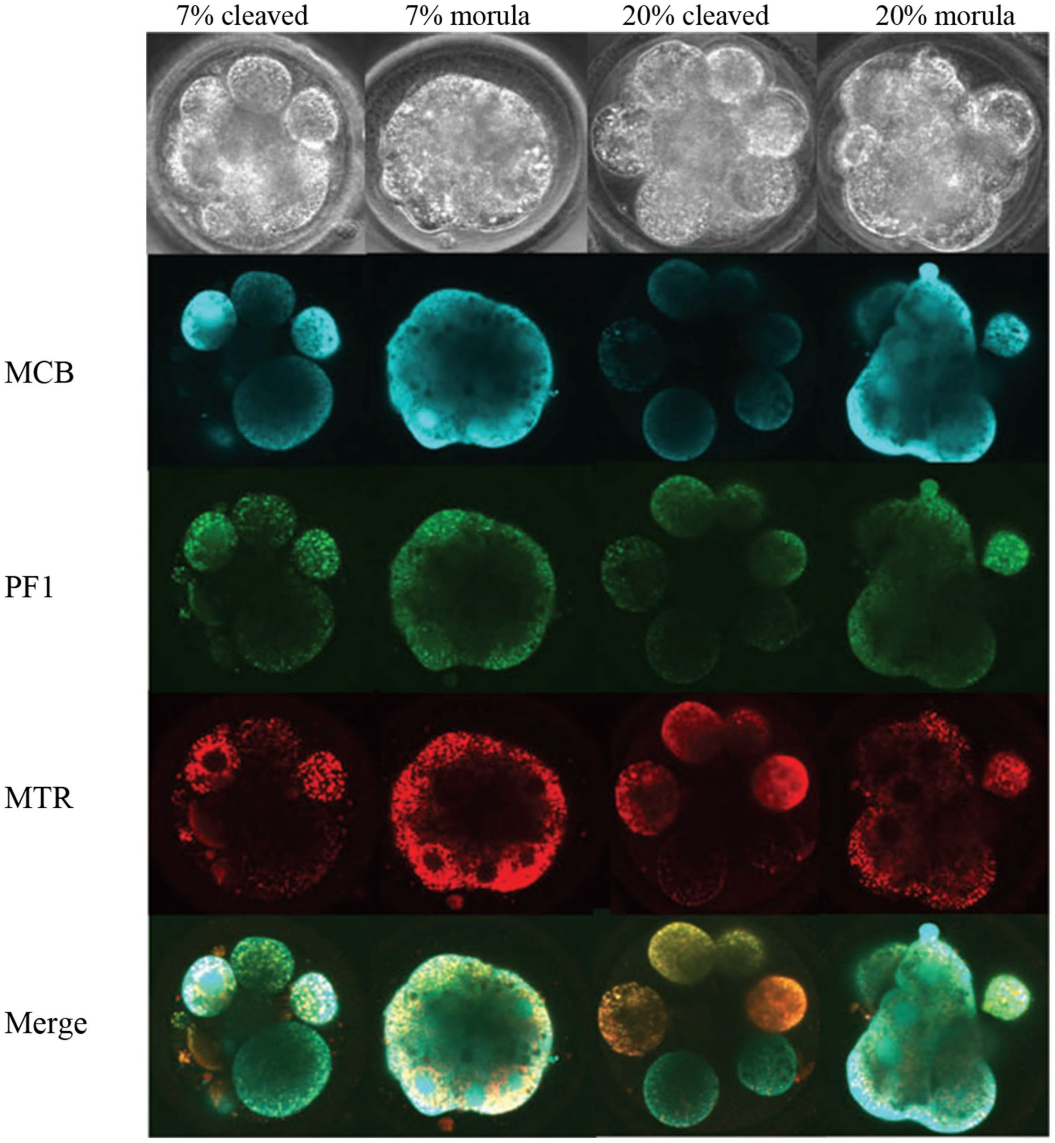
Images of Day 5 (cleaved) and on-time (morula) embryos cultured in 7 and 20% O2 and labeled with fluorescence probes (33). MCB, monochlorobimane (reduced glutathione); PF1, perfluoxy 1 (hydrogen peroxide) and MTR, Mitotracker Red CMXRos (active mitochondria).

Sutton-McDowall et al. (33) evaluated the effect oxygen concentration (7% = optimal vs. 20% = stressed) in incubators on metabolic heterogeneity of the bovine embryos using hyperspectral microscopy. The embryos were exposed to two oxygen concentrations for 5 days (metabolism measurements) or for 8 days (for embryo developmental competence), and imaged (Figure 3). It was reported that exposure to 20% oxygen following fertilization reduced total, expanded and hatched blastocyst rates by 1.4, 1.9 and 2.8 fold, respectively, compared to a 7% oxygen environment.

Santos et al. (34) used hyperspectral imaging to characterize the metabolites produced by bovine embryos during development (on-time development or fast-development). The metabolic activity and DNA damage for the on-time developing embryo (Day 2: 2 cell; Day 4: 5–7 cell) and fast-developing embryo (Day 2: 3–7 cell; Day 4: 8–16 cell) were analyzed. The Hyperspectral microscopy was used to assess a broader range of endogenous fluorophores. They measured the DNA damage using ŸH2AX immunohistochemistry. It was found that fast-developing embryos showed lower abundance of endogenous fluorophores (lower metabolic activity) than that of on-time embryos on Day 2. The fast-developing embryos showed a ‘quiet’ metabolic pattern on Day 2 and Day 4 of development, than that of on-time embryos. Also no difference was found in the level of DNA damage between on-time and fast-developing embryos on either day of development.

## 7 Other techniques

A microfluidic chip method was developed by Szczepanska et al. (35) to evaluate bovine oocyte and embryo. In the preliminary trials, they used a miniature spectrophotometric system in the microfluidic chip, and reported that this custom-built lab-on-chip has the potential to determine IVF results and bovine pregnancy rate.

## 8 Prospects and future direction

1. In the last decade lots of work have been done on human embryos using morphokinetics or time-lapse imaging or other imaging approaches. The advancements in artificial intelligence techniques including machine learning has been efficiently incorporated to develop high accuracy classification models to predict the quality of human embryos. However, in general, the research on bovine embryo quality assessment is very limited. As first step, the successful approaches and tools in human study should be investigated for bovine embryos with appropriate modifications.
2. More research is warranted for bovine embryos using color /optical microscopy supported with ML based image analysis and classification tools. These techniques offer simple operational procedures, no damage to live cells, and easy to implement.
3. The potential of non-morphological features in bovine embryo quality assessment should be thoroughly studied with imaging or spectroscopic techniques such as Raman spectroscopy/ imaging, FTIR, NIR hyperspectral imaging and so on. Cofactors other than morphological features such as timing of first embryonic cell divisions or metabolic profiles may be used to predict the ability of an embryo to establish pregnancy or as markers of embryonic viability. In general, more metabolomics profile of embryos should be established for bovine subjects.

## 9 Conclusion

In the current manual grading of bovine embryos, only qualitative morphological features are used for decision making. However, while using image based grading systems, additional data such as proteomics, metabolomic profile, quantitative or constructed morphological features can be incorporated to obtain beneficial information about embryos (36). Researchers are currently in pursuit of developing an accurate, repeatable, and most importantly, objective method of determining the gestational success of bovine embryos post-transfer. More research is warranted on morphokinetics of bovine embryos, metabolomics profiles of bovine embryo culture media, and composition of bovine embryos including nucleic acids and amides to build a database for developing robust models to deploy image or spectroscopy based techniques in commercial facilities.

## Author contributions

MS: Writing – original draft, Writing – review & editing. PM: Writing – review & editing.

## Footnotes

^1^ http://blasto3q.com
